# Gaining control over complex distal radius and ulna fractures: tips and tricks

**DOI:** 10.1308/rcsann.2023.0069

**Published:** 2023-10-16

**Authors:** M Kammela, D Biswas, K Karuppaiah

**Affiliations:** King’s College Hospital NHS Foundation Trust, UK

## BACKGROUND

**Figure 1:**
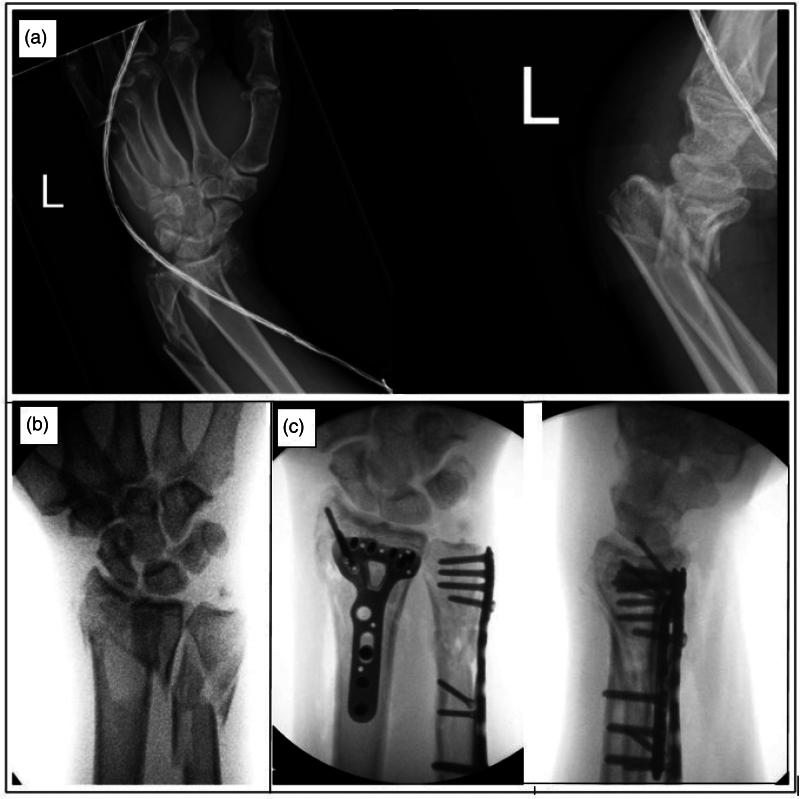
Radiographs of the wrist. (a) Anteroposterior and lateral views of the wrist at presentation. (b) Wrist in traction in wrist tower. (c) Lateral and anteroposterior views of the wrist on completion of surgery.

The restoration of anatomy in complex comminuted distal radius and ulna fractures is essential for regaining wrist movement, forearm rotation and preventing arthritis.^1^ However, achieving the correct length and rotational alignment of the radius and ulna can be challenging in cases of severe comminution (Figure 1a). Traditional fixation methods often make it difficult to obtain suitable posteroanterior and lateral views, because the wrist needs to be rotated constantly to achieve effective control over fragmented bone segments during fixation.^2^ Although external fixation/infix has been recommended for these injuries, they come with increased risks of complex regional pain syndrome, malunion,^3^ wrist stiffness and the need for further surgeries. The use of wrist traction with finger traps has been described for closed manipulation and reduction of distal radius fractures in the emergency department and arthroscopy-assisted fixation of wrist fractures.^4,5^ Utilisation of a wrist traction tower overcomes the disadvantages of conventional traction techniques by providing traction and alignment in neutral rotation during fixation. This is the first time this technique has been described in detail for the surgical management of complex wrist fractures.

## TECHNIQUE

**Figure 2:**
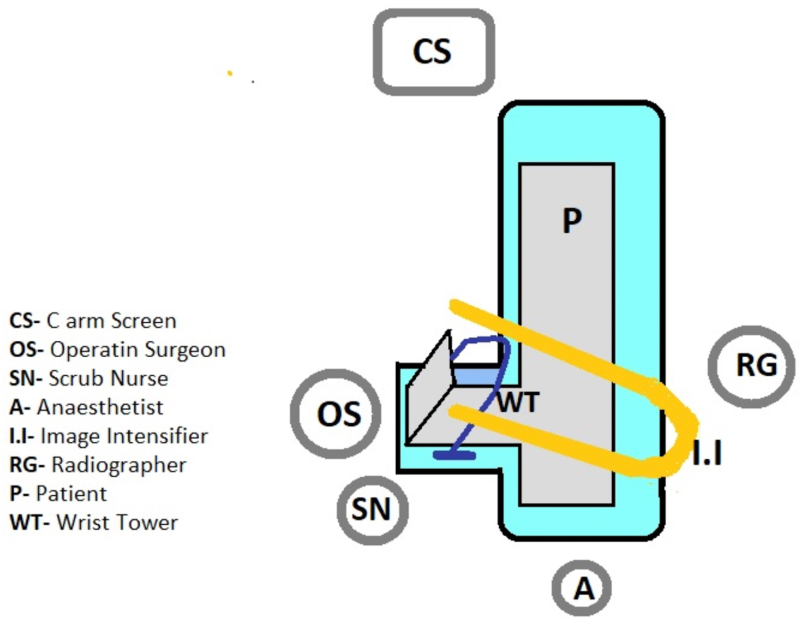
Orientation of the surgeon, theatre staff and image intensifier with respect to the patient and wrist tower. The image intensifier is positioned on the patient’s uninjured side and the C-arm is rotated 90° to the vertical and brought in across the patient.

The patient is supine with the arm extended over an arm board, and the image intensifier and staff are positioned as shown in Figure 2.

**Figure 3:**
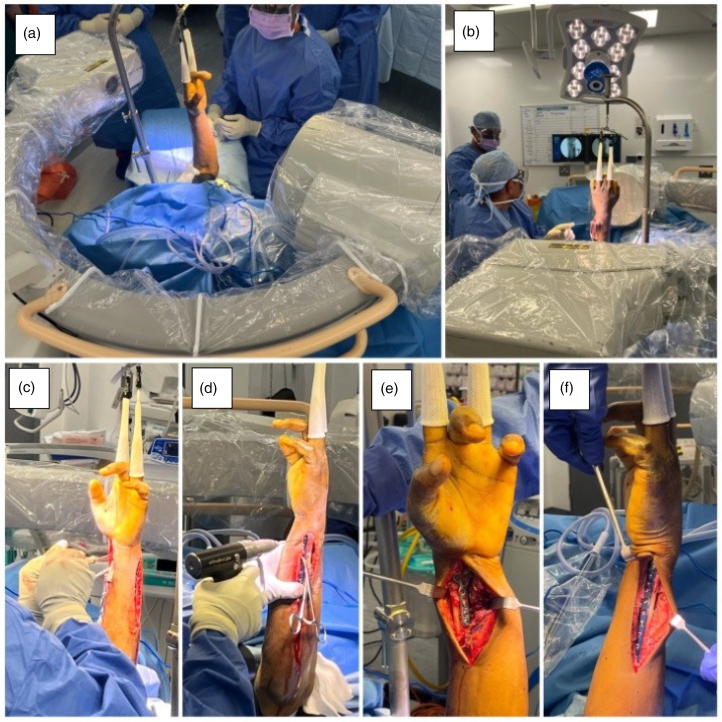
The patient was positioned with the wrist in traction in a wrist tower. ( a) Position of the patient’s wrist in the wrist tower with respect to the surgeon. (b) Position of the patient and the set-up with respect to the image intensifier screen. (c,d) Simultaneous approach to the radius and ulna. (e) Final image of radius plating. (f) Final image of ulna plating.

The shoulder is abducted to 90°, the elbow is flexed to 90° and the forearm is in a neutral position with 10lb of traction applied under tourniquet control. This allows for proper wrist alignment and orthogonal views during fixation (Figure 1b). The image intensifier is positioned on the opposite side, providing unobstructed access to the wrist for the surgeon and scrub team (Figures 2 and 3a,b). The ulna is approached through the subcutaneous border and fixed with lag screws and a posterolateral ankle plate. The distal radius fracture is approached with a volar Henry approach and fixed with a distal radius plate (Acumed) (Figure 3e).

## DISCUSSION

Treating complex comminuted fractures of the distal radius and ulna can be a difficult task. However, our innovative technique addresses the technical challenges commonly encountered with traditional fixation methods. Our approach provides improved control over fragment rotation, unrestricted access to the fracture site and the ability to obtain orthogonal views, resulting in more successful outcomes.
